# Picture of Better Health: Prioritizing Air Pollution Control in China

**DOI:** 10.1289/ehp.118-a398b

**Published:** 2010-09

**Authors:** Cynthia Washam

**Affiliations:** **Cynthia Washam** writes for *EHP*, *Oncology Times*, and other science and medical publications from South Florida

China’s Yangtze River Delta is one of the most heavily polluted and densely populated areas of the world, with some districts of Shanghai exceeding a population density of 40,000 people/km^2^. Annual average concentrations of fine particulate matter (PM_2.5_), microscopic airborne particles that cause heart and lung damage, are estimated to vary from 14 μg/m^3^ in the cleanest areas to 133 μg/m^3^ in the dirtiest. By contrast, most major cities in the United States have annual average PM_2.5_ levels between 7 and 20 μg/m^3^. Yet a new study suggests regions as highly polluted as the Yangtze River Delta can clear the air and in the process save thousands of lives by adopting common pollution-control technologies **[*EHP*** **118(9):1204–1210; Zhou et al.]**.

The study is believed to be one of the first risk assessments of a developing country using the Community Multiscale Air Quality (CMAQ) modeling system. Although CMAQ is widely used in the United States, most developing countries lack the exhaustive data on emissions, pollutant monitoring, and weather conditions needed to make accurate predictions. The research team capitalized on emissions data from China that NASA estimated using satellite measurements during a 2006 study of intercontinental pollution drift.

The researchers examined emissions of sulfur dioxide (SO_2_), nitrogen oxides (NO_x_), volatile organic compounds (VOCs), and PM_2.5_, focusing on the effect of these pollutants on PM_2.5_ and ozone concentrations. Ozone is a gas formed when NO_x_ and VOCs in the atmosphere react in sunlight. PM_2.5_ is linked to heart and respiratory disease, with resulting effects on premature death, and ozone has been associated with respiratory outcomes and premature death. They considered 10 control scenarios among major pollution sources—industry, power plants, motor vehicles, and domestic life—and focused on the health benefits per ton-of-emission reduction across scenarios.

Computer modeling predicted the greatest decline in mortality would come from reducing SO_2_ emissions from power plants and PM_2.5_ emissions from industry, with each technology preventing approximately 1,200–24,000 deaths per year among the more than 80 million people living in the delta. This is based on the significant reduction in SO_2_ emissions from coal-powered plants with the use of fluidized gas desulfurization, as well as the large health benefit per ton of avoided emissions of primary PM_2.5_ from industry. Measures to reduce NO_x_ and VOCs, including tighter vehicle-emissions standards, would have less impact on public health.

The authors point out that understanding sectoral differences in the ways emission-control strategies affect exposures and health risks can help guide strategies that are both economically and environmentally optimal. They conclude that their findings provide the basis for prioritizing pollution-control strategies in the Yangtze River Delta and provide a template for comparable analyses elsewhere.

